# Effectiveness of seasonal influenza vaccine in elementary and middle schools: a 10-year follow-up investigation

**DOI:** 10.1186/s12879-022-07898-y

**Published:** 2022-12-06

**Authors:** Teruyuki Kajiume, Sumera Mukai, Nobutaka Toyota, Ikuo Kanazawa, Akiko Kato, Etsushi Akimoto, Toshio Shirakawa

**Affiliations:** 1 Hiroshima Akichiku Medical Association, 5-13 Sakae-machi, Kaita-cho, Aki-gun, Hiroshima, 736-0043 Japan; 2 Mukainada Child Clinic, 24-26 Aosaki-naka, Fuchu-cho, Aki-gun, Hiroshima, 735-0016 Japan; 3 Mukai Clinic of Internal Medicine, 2-2-8 Tahara, Ondo-cho, Kure-city, Hiroshima 737-1216 Japan; 4 Toyota Ladies Clinic, 4-30-1 Kawasumi, Kumano-cho, Aki-gun, Hiroshima, 731-4223 Japan; 5 Kanazawa Cardiology Clinic, 4-10-18 Yano-nishi, Aki-ku, Hiroshima, 736-0085 Japan; 6 Kato Gastroenterology Clinic, 3-3-14 Nakano-higashi, Aki-ku, Hiroshima, 739-0323 Japan; 7 Akimoto Clinic, 3-34 Inari-machi, Kaita-cho, Aki-gun, Hiroshima, 736-0067 Japan; 8 Senosirakawa Hospital, 1-28-3 Seno, Aki-ku, Hiroshima, 739-0311 Japan

**Keywords:** Follow-up studies, Surveys and questionnaires, Influenza vaccines, Immunization, Infection

## Abstract

**Background:**

Influenza spreads from schools to the rest of society. Thus, we conducted questionnaire surveys of influenza vaccination in elementary and middle schools in a district for 10 years to determine immunization rates and infection conditions among students who were potential sources of infection at home.

**Methods:**

The questionnaire-based survey on influenza vaccine administration, influenza infection, and influenza types contracted, as well as influenza immunization history, was conducted in 10 seasons over a period of 10 years.

**Results:**

In elementary schools, vaccination was associated with lower morbidity in most years, whereas in middle schools, morbidity increased among students who were vaccinated every year. Our study did not find consistent trends among faculty and staff. In addition, we found that morbidity was significantly higher among elementary (*P* < 0.001) and middle (*P* < 0.05) school students who had been vaccinated since infancy than among those who had not been vaccinated since infancy.

**Conclusions:**

The results of this study suggest that vaccinating infants for influenza may increase the risk of contracting influenza later in life.

**Supplementary Information:**

The online version contains supplementary material available at 10.1186/s12879-022-07898-y.

## Background

Influenza is an acute viral disease caused by an influenza virus infection. Typical influenza often presents with acute high fever, upper respiratory tract symptoms, such as cough and runny nose, and muscle pain. Complications include pneumonia and influenza encephalopathy, which may lead to death. The World Health Organization recommends annual influenza vaccination for all people aged ≥ 6 months, especially for those at a higher risk of contracting this disease [1]. According to the United States Centers for Disease Control and Prevention, vaccine effectiveness (VE) for all age groups was 39% in the 2019–2020 season. In the last decade, the effectiveness was approximately 30–50% in most years but was particularly low (19%) in the 2014–2015 season [2].

The influenza vaccine used in Japan is a quadrivalent inactivated influenza vaccine (trivalent vaccine until 2014), which is a split vaccine based on hemagglutinin (HA) [3]. Split vaccines are highly safe; however, their signals have difficulty transmitting through the innate immune system because they are based on HA protein and thus may not be effective in children with no history of influenza infection [4].

The Japanese influenza vaccine is made following the World Health Organization recommendations for strains that are announced annually. In Japan, the influenza vaccine strain for each season is standardized among manufacturers, and two influenza vaccine doses are recommended per season for children aged < 12 years (mostly elementary school students) and one dose for everyone else (middle school and older). In general, young children with little or no history of infection are prone to influenza, and infections may spread from the school-going children to the rest of society. An extended observation period allows us to assess trends that are difficult to analyze with data from only a few influenza seasons. In the last decade, we conducted questionnaire surveys of influenza vaccination among children, students, teachers, and staff at schools in a district to understand influenza immunization rates (IRs), infection conditions, and VE in schools that can be sources of infection. This article summarizes some of the interesting findings that we have observed over the past 10 years.

## Methods

The participants were students and staff from 38 elementary schools and 23 middle schools in an area covered by the Akichiku Medical Association. The survey was conducted over 10 seasons of influenza from 2010–2011 to 2019–2020. The number of participants surveyed per season included a minimum of 9047 (2015–2016 season) to a maximum of 12,002 (2011–2012 season) elementary school students, a minimum of 4034 (2015–2016 season) to a maximum of 5491 (2016–2017 season) middle school students, and a minimum of 1034 (2015–2016 season) to a maximum of 1392 (2016–2017 season) faculty and staff.

The legal guardian or parent was responsible for answering the questionnaire for students, whereas staff members were responsible for answering their questionnaires. The questionnaires were anonymized. The parents/guardians were requested to answer questions such as: “Did you get the influenza vaccine?” “Did you contract influenza?” and “If so, what type of influenza did you contract?” The questionnaire data were collected once a year before summer. The questionnaire response rate was 88.03 ± 4.01%.

The participants were vaccinated by regular doctors or other physicians. As researchers, we did not interfere with the location and time of vaccination. The participants were classified as unvaccinated or completely vaccinated. Participants who reported receiving the recommended dose during the target season were categorized as “completely vaccinated,” while those who were not vaccinated once in that season were categorized as “unvaccinated.” Participants who were partially vaccinated, such as an elementary school student who should have received two doses but only received one—7486 among 46,547 (16.08%) students over the 10 years—were excluded. The current gold standard for VE is a test-negative case-control study design (TNCC) [5–7]; however, this was not possible with our surveys. Therefore, we used the chi-square test for the analysis of VE in the vaccinated and unvaccinated participants, at a significance level of 5% (*P* < 0.05). The rate of immunization was calculated as follows: *number of vaccinated participants/number of participants who responded to surveys × 100 (%)*. Morbidity was calculated as follows: *number of disease onsets/sum of vaccinated or unvaccinated persons × 100 (%).* VE is concerned with relative risk reduction. The concept was first proposed by Yule and Greenwood in 1915 to elucidate the efficacy of the typhoid and cholera vaccines [8]. The VE was determined as follows: *(morbidity rate of unvaccinated−morbidity rate of vaccinated)/morbidity rate of unvaccinated × 100 (%).* The IR was determined as follows: *vaccinated/(vaccinated + unvaccinated) × 100 (%).* Non-effectiveness was defined as cases of influenza despite vaccination for the particular season. BellCurve for Excel (Social Survey Research Information Co., Ltd., Tokyo, Japan) was used for the analysis. Finally, in the last year, the following question was added to the questionnaire: “Have you been receiving the influenza vaccine since infancy?”.

## Results

Table 1 shows the IR over 10 years, starting in 2010. A high IR of approximately 50% was observed among elementary and middle school students in 2010–2011 and 2011–2012, possibly because of the novel influenza epidemic in 2009–2010. Thereafter, the IR gradually declined to 30–40% in 2019–2020. The IR of faculty and staff remained between 30% and 40% over the 10 influenza seasons.

**Table 1:** Immunization rate and vaccine effectiveness

			2010–2011	2011–2012	2012–2013	2013–2014	2014–2015	2015–2016	2016–2017	2017–2018	2018–2019	2019–2020
Elementary school	IR	(%)	54.7	51.2	46.7	48.8	46.0	43.8	40.9	34.9	39.3	43.4
	VE	(%)	10.1	10.4	5.5	26.4	3.7	15.8	16.7	26.6	21.8	– 5.6
		n =	9803	9622	9899	10,225	9706	9630	10,449	10,763	10,781	10,190
			*P* < 0.001	*P* < 0.01	*P* = 0.061	*P* < 0.001	*P* = 0.235	*P* < 0.001	*P* < 0.001	*P* < 0.001	*P* < 0.001	*P* = 0.044
Middle school	IR	(%)	49.4	50.6	42.3	41.3	37.8	37.4	37.4	32.8	34.6	34.8
	VE	(%)	– 18.5	– 15.9	–18.5	– 32.4	– 45.6	– 3.6	– 11.7	– 8.7	– 27.9	– 29.6
		n =	4633	4315	4204	4605	4296	3763	5144	4653	4749	4111
			*P* < 0..001	*P* < 0.01	*P* < 0.01	*P* < 0.001	*P* < 0.001	*P* = 0.494	*P* = 0.025	*P* = 0.055	*P* < 0.001	*P* < 0.001
School staff	IR	(%)	37.4	37.2	31.3	3.7	35.8	35.9	35.2	33.1	38.2	42.2
	VE	(%)	– 37.8	22.2	39.4	–9.2	– 27.0	– 7.2	11.9	– 2.3	2.9	– 6.4
		n =	1211	937	996	1112	881	925	1153	1065	1042	1027
			*P* = 0 .108	*P* = 0.286	*P* < 0.01	*P* = 0.692	*P* = 0.212	*P* = 0.705	*P* = 0.455	*P* = 0.888	*P* = 0.888	*P* < 0.01

*IR* immunization rate; *VE* vaccine effectiveness

**Fig. 1:**
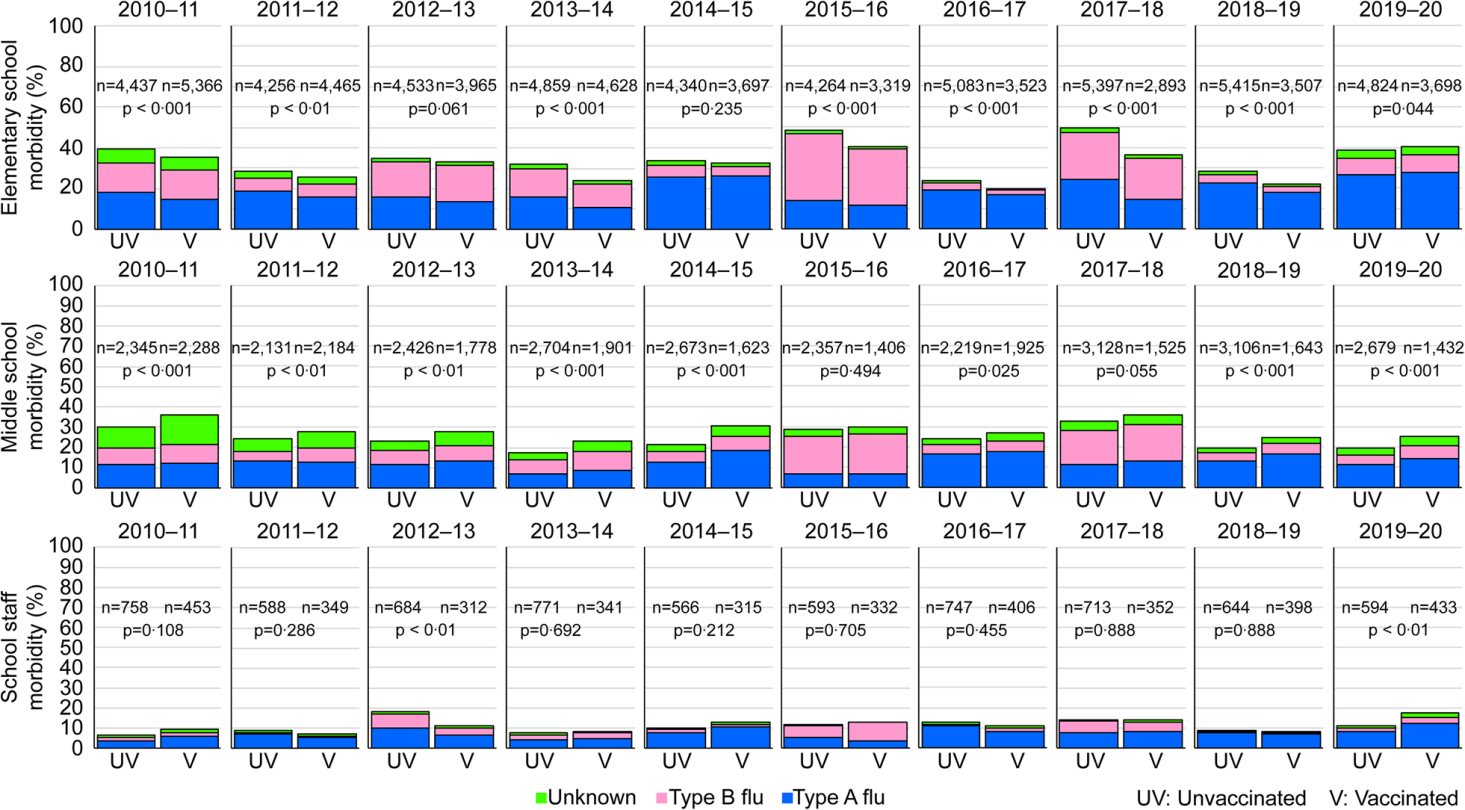
Comparison of morbidity between the vaccinated and unvaccinated groups. In elementary school students, morbidity in the vaccinated group was significantly lower during the 10-year period, except during 2012–2013, 2014–2015, and 2019–2020. Among middle school students, morbidity was higher among the vaccinated students every year. A consistent trend in morbidity was lacking among faculty and staff.

Morbidity was compared between the vaccinated and unvaccinated groups (Fig. 1). In elementary school students, morbidity was lower in the vaccinated group in all surveyed seasons except in 2019–2020, and significant differences were found in seven seasons (no significant difference in the years 2012–2013 and 2014–2015) between the vaccinated and unvaccinated groups. However, among middle school students, morbidity was not lower in the vaccinated group than in the unvaccinated group in any of the 10 seasons. In fact, morbidity was significantly higher among the vaccinated middle school students than among the unvaccinated middle school students in 8 of the 10 years. The difference was not significant in the years 2015–2016 and 2017–2018. Among faculty and staff, there were no significant differences in 8 of the 10 years; within the vaccinated group, morbidity was significantly lower in 2012–2013 but was significantly higher in 2019–2020.

The performance of a vaccine is often expressed as VE, and Table 1 shows VE recorded in each influenza season. In elementary school students, VE was the highest (26.6%) in 2017–2018 and was generally around 10–20%. Among middle school students, the vaccine was not effective in any of the 10 influenza periods and was the lowest (− 45.6%) in 2014–2015. A consistent trend was lacking among the faculty and staff. The data used to create Fig. 1 are provided in Additional file 1.

**Fig. 2:**
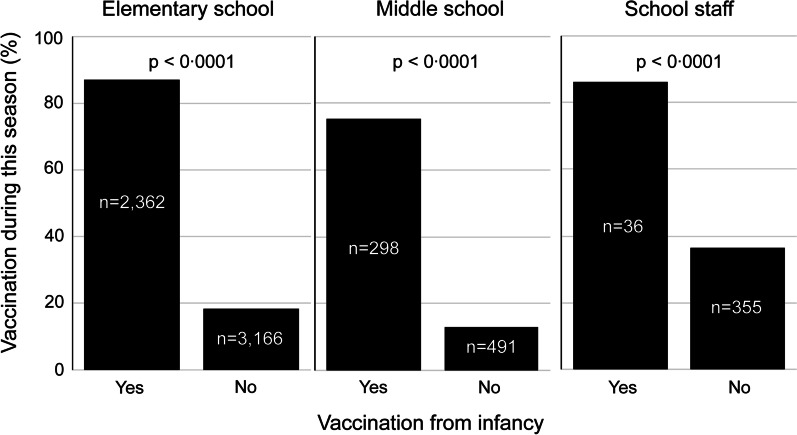
Relationship between influenza vaccination from infancy and vaccination in 2019–2020. Elementary school students, middle school students, and faculty and staff were likely to be vaccinated against influenza in the last season if they had been vaccinated in infancy

**Fig. 3:**
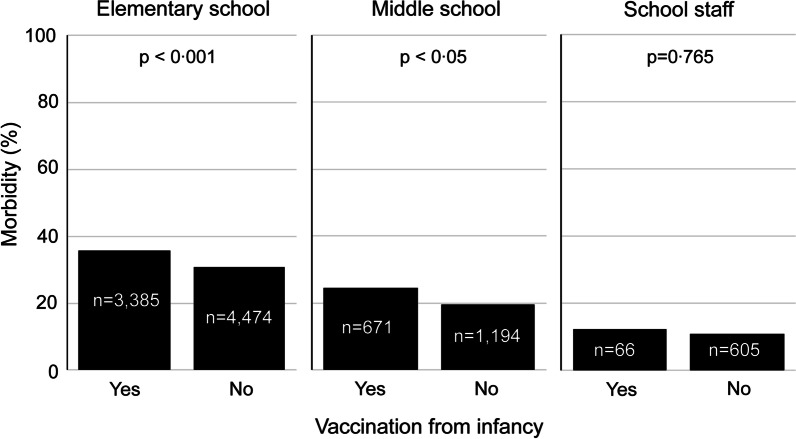
Relationship between morbidity and vaccination from infancy. Influenza morbidity was significantly higher in elementary and middle school students who had been vaccinated since infancy compared with those who were unvaccinated since infancy. The difference among faculty members and staff was not significant

Among all participants, the proportion of the participants who had been vaccinated since infancy was higher among those who were vaccinated in 2019–2020 (Fig. 2). Elementary and middle school students who had been vaccinated since infancy had a significantly higher influenza morbidity (elementary students, *P* < 0.05; middle school students, *P* < 0.05); however, the differences among staff were not significant (Fig. 3).

## Discussion

According to a 2003 report, influenza VE was 68% and 85% among 0–15-year-old individuals who received one and two doses, respectively. In addition, VE was 55% and 82% among 16–64-year-old individuals who received one and two doses, respectively [9]. Recent studies of children aged 6 months to 15 years found that VE was 38–68% for influenza type A and 26–39% for influenza type B [10–12]. The present survey by our medical association showed a VE of 10–20% among elementary school students. The vaccines were not effective for middle school students in the 10 years, and no consistent trends were found among faculty and staff. Our findings differ greatly from those generally reporting preventive effects of influenza vaccines. Morbidity was higher in this study, probably because we examined schools, which might be centers of influenza epidemics, than in other studies that did not include participants from the school setting. It is difficult to define “development of severe disease” in influenza, and a survey to determine the number of cases of complicated pneumonia and encephalitis associated with influenza would be of questionable reliability. We, therefore, did not investigate whether the vaccines were able to suppress severe disease. Notably, Ritzwoller et al. found that in children aged 6 months to 8 years, the effectiveness of a two-dose vaccine against influenza-like illness and pneumonia was 23% and 51%, respectively, and a two-dose vaccine was significantly better than a one-dose vaccine (23%) in improving pneumonia [13].

The surprising finding that morbidity was higher among middle school students who were vaccinated is likely related to vaccination from infancy. A TNCC study that examined influenza VE by age in three consecutive seasons in Japan found that VE was poor in children aged < 1 year and was highest in those aged 1–5 years, thereafter decreasing with age [14]. This finding suggests that influenza vaccination should not be strongly recommended for children aged 6–11 months. Notably, our study did not include infants, and we did not use the TNCC method. Despite these differences, our results showed similar trends. We considered three possible reasons for the unexpected phenomenon of higher morbidity among vaccinated middle school students. First, unlike elementary school students, middle school students were only vaccinated once. However, while this hypothesis may explain a decrease in VE, it does not explain why vaccinated students had higher morbidity. Second, children from families who do not intend to vaccinate may go undiagnosed because they might not seek medical care even if they contract influenza. Japan has a national health insurance system that makes it easy for people to visit clinics [15]. In addition, schools recommend that students be examined by a doctor, even for mild symptoms. Thus, because it is highly likely students would be examined even for mild symptoms; the impact of this hypothesis on the findings of this study is believed to be very small. Third, students from families that vaccinate their children against influenza in middle school may have been vaccinating them since infancy. Parents who seriously vaccinate their junior high school children as recommended have probably had their children vaccinated since infancy. In other words, because of the “original antigenic sin” [16–18] that is observed with split vaccines, these children may have difficulty building immunity to future influenza viruses.

The host’s initial response to microbial infection is mediated by innate immunity. For influenza viruses, viral double-stranded RNA (dsRNA) that enters the cytoplasm is recognized by receptors of the retinoic acid-inducible gene-I family [19]. Extracellularly, toll-like receptor (TLR) 3 detects the viral dsRNA [20, 21] and TLR7 detects the single-stranded RNA [22–24] to induce inflammatory cytokines, such as interferon. Adaptive immunity is acquired after these initial responses to infection. Influenza vaccines in Japan are split vaccines based on HA proteins that have superior safety. Because their signals do not enter the innate immune system, they are effective for people who have already been infected with influenza. Split vaccines, however, cannot be expected to be effective for naïve individuals with no history of influenza infection [4]. If a naïve individual is vaccinated with an HA protein product before they contract an influenza virus derived from the wild strain, it is possible that the effect will be rather weak. However, because there is a small immune response, the phenomenon of “original antigenic sin” may arise due to common antigens present besides the HA protein of the vaccine strain. The original antigenic sin refers to the immunological imprinting of the first viral infection encountered; however, when the person is later infected with another type of influenza, the immune system is unable to respond, regardless of its immunogenicity [25]. This phenomenon was first reported in 1947 [26]. The influenza virus comprises 10 proteins synthesized from an 8-segmented RNA genome [27, 28]. The original antigenic sin may be responsible for the ability to acquire immunity for the 9 non-HA structural proteins in the Japanese population because split vaccines are used in Japan. Thus, when infected by a subtype different from that of the initial vaccine, the desired effect may not be achieved.

It has been reported that VE decreases dramatically among people who receive the influenza vaccine every season [29]. While the mechanism of this negative effect from repeated vaccination remains unclear, several hypotheses mention the original antigenic sin. For example, Hoskins et al. concluded that repeated vaccination with inactivated vaccines had no long-term benefits in preventing infection from influenza viruses that caused epidemics in the 1970s [30]. The “antigenic distance hypothesis” states that the decline in effectiveness from repeated vaccination is due to differences in the antigenic distance between the vaccine strain and epidemic strain. This hypothesis describes a phenomenon in which the adverse effects of repeated vaccination appear when the current vaccine and previous vaccine are antigenically similar, although the current vaccine strain and epidemic strain are different [31, 32]. Because this study only comprised a questionnaire-based survey of school children, a comparison between vaccine strains and epidemic strains was beyond the scope. Thus, we could not determine the participants’ internal immune responses. Such an investigation could help elucidate our study observations better.

## Conclusions

The results of this study indicate that vaccinating infants for influenza may increase the risk of contracting influenza later in life. However, further research is needed, especially regarding the development of severe disease. Therefore, we cannot recommend halting influenza vaccinations for infants based solely on the findings of this study.

## Supplementary Information

**Additional file 1**: Detailed numbers of the participants over the 10-year study period. The results of the questionnaire for the 10 years after the 2010–2011 season, tabulated by grade. The status of influenza infection can be viewed by vaccination and non-vaccination. 

## Data Availability

All data generated or analyzed during this study are included in this published article and its Additional file 1.

## Abbreviations

VE: Vaccine effectiveness
HA: Hemagglutinin
IR: Immunization rate
TNCC: Test-negative case-control study design
dsRNA: Double-stranded RNA
TLR: Toll-like receptor
